# Recommendations to improve physical activity prescription for the cystic fibrosis population: an Irish perspective

**DOI:** 10.1186/s12913-020-05910-2

**Published:** 2020-11-19

**Authors:** Nicola Hurley, Bróna Kehoe, Noel McCaffrey, Karen Redmond, Lydia Cullen, Niall M. Moyna

**Affiliations:** 1 School of Health and Human Performance, Dublin City University, Dublin, Ireland; 2 Department of Sport and Exercise Science, Waterford Institute of Technology, Waterford, Ireland; 3 ExWell, Dublin, Ireland; 4 Mater Misericordiae University Hospital, Dublin, Ireland; 5 Beaumont Hospital, Dublin, Ireland

**Keywords:** Clinical education, Clinical practice, Physical activity prescription, Healthcare professional, Physical activity, Health promotion, Optimize patient outcomes, Cystic fibrosis

## Abstract

**Background:**

Physical activity (PA) is a well-established therapeutic modality for the maintenance and improvement of long-term health in cystic fibrosis (CF). Healthcare professionals (HCP) are considered credible and well-placed messengers for the delivery of PA advice. Limited research exists investigating the extent of PA prescription within CF care. This study aimed to identify Irish HCP i) knowledge and practice of, and ii) motivators and barriers to PA prescription, and iii) proposed strategies to optimize PA promotion and prescription in CF populations.

**Methods:**

HCP from six designated CF centres in Ireland and members of the national physiotherapy CF clinical interest group were invited to participate. Following an expression of interest, each HCP (*n* = 81) received an email containing the plain language statement and link to the online survey. 48 HCP (physiotherapists *n* = 24, other n = 24) completed the 30-item investigator-developed survey, which included multiple choice single answer, matrix style and open-ended questions.

**Results:**

Most HCP (81%) acknowledged that discussing PA with CF patients was part of their professional role. Almost all physiotherapists (95%) reported having sufficient knowledge regarding PA prescription, compared to 17% of other HCP. All physiotherapists reported discussing PA at every patient interaction, with 81% employing the current consensus guidelines, compared to 33 and 5% of other HCP, respectively. Among the most common barriers reported by HCP to recommending PA to their CF patients were; lack of motivation and compliance among patients to adhere to PA advice, limited availability of PA programmes to refer their patients to, limited time with patients during clinic visits and a lack of knowledge regarding PA prescription for CF care. Three-quarters of HCP reported a need to improve PA services for CF patients in Ireland.

**Conclusion:**

As people with CF are living longer, it is imperative that HCP are expanding their scope of practice to include discussions around PA at every patient visit. Formal educational opportunities in the form of continuing professional development programmes are warranted for CF HCP to optimize long-term patient management and outcomes. There is also a need to develop patient-centered and evidence-based PA programmes underpinned by theories of behaviour change to enhance motivation and compliance among CF patients.

**Supplementary Information:**

The online version contains supplementary material available at 10.1186/s12913-020-05910-2.

## Background

Cystic fibrosis (CF) is an autosomal recessive disorder characterized by the abnormal functioning of the CF transmembrane conductance regulator (CFTR) protein that is essential for the regulation of transmembrane chloride reabsorption [1]. It is a multisystem disorder involving abnormal function of chloride channels in secretory epithelial cells lining the airways, digestive system, reproductive system, and the skin and results in increased morbidity and mortality [2]. The prevalence of CF differs by ethnicity and geographical background. It is the most common inherited disease among the Caucasian population, with an annual incidence of approximately 1 in 2500 live births [3]. Ireland has the highest incidence per capita in the world, with approximately 1 in every 19 people carrying the CF mutation and 7 in every 10,000 people living with the condition [4].

The life expectancy of CF patients has substantially lengthened in the past 25 years due to early diagnosis and improvements in symptomatic therapeutic regimens [5]. Optimal management of CF involves a multidisciplinary team (MDT) of health care professionals (HCP) and is centered around slowing lung function deterioration, providing dietary interventions to compensate for pancreatic insufficiency and intestinal malabsorption and optimizing pharmacotherapy to eliminate infections [6].

Exercise intolerance is a hallmark of CF disease. Evidence suggests that children with CF tend to have similar PA levels to their healthy peers until they reach adolescence where there is a notable decline. Cox et al., report that this decreased activity in adolescents with CF does not recover with increasing age [7]. Participation in regular PA has been shown to improve physical deconditioning in both adults and children with CF [8, 9]. Among CF patients, regular physical activity (PA) has the potential to decrease the annual rate of decline in pulmonary function, improve airway clearance, reduce hospitalization frequency and improve longevity, while also improving aerobic capacity, muscle strength, bone health and enhancing health-related quality of life [10, 11].

HCP are considered desired, credible and well-placed messengers for the delivery of PA advice and have the capacity to play an integral role in the promotion of PA behaviours among their patients [12, 13]. There is a greater likelihood of patient engagement in PA behaviours following pro-active counseling by HCP [14, 15]. According to the American College of Sports Medicine *Exercise is Medicine* initiative, all HCP should incorporate discussions about PA into *every* patient interaction [14]. Therefore, it is imperative that HCP work collectively within the multidisciplinary team to ensure their PA recommendations are underpinned by both up-to-date and evidence-based theory. Expert consensus guidelines have been developed for use by CF HCP responsible for discussing and prescribing PA with their patients [16].

It is critical that every member of the MDT strives to maintain the same philosophy about the importance of PA, particularly for individuals with chronic disease, making every contact count. Although the primary responsibility for PA prescription currently lies with the physiotherapist, it is imperative that other members within the MDT positively reinforce PA prescription with evidence-based PA promotion.

PA prescription is considered to be synonymous with any other form of prescription, including a type, dose, frequency, duration and therapeutic goal [17]. PA prescription involves careful screening including the patient’s capacity for PA, as well as a needs analysis to identify the individual’s goals and interests.

To our knowledge, currently no data exist on HCP practice of PA prescription within CF care.

The purpose of this study was to investigate the knowledge and practice surrounding PA promotion and prescription among CF HCP in Ireland. Specifically, the study evaluated i) the current level of knowledge regarding CF-specific PA guidelines and prescription, ii) the practice of discussing and appropriately prescribing PA, iii) the motivators and barriers to recommending PA, and iv) strategies to optimize HCP prescription of PA to patients with CF.

## Methods

### Participants

HCP working in hospitals and specialist CF centres were invited to participate in the study. The term healthcare professional, within the context of this study, refers to medical and allied HCP working in CF care. In Ireland, the CF MDT is typically comprised of consultants, registrars, clinical nurse specialists, dieticians, physiotherapists, psychologists, social workers and pharmacists. Historically, in Ireland, exercise-related programmes have been delivered by physiotherapists, as the role of the exercise scientist does not yet have professional recognition within the Health Service Executive.

### Recruitment and survey dissemination

Participants were recruited via two routes: i) direct contact with clinical CF teams, and ii) through the national physiotherapy CF clinical interest group. The email address and/or telephone number for eleven of the designated CF centres in Ireland were obtained from the Cystic Fibrosis Ireland website. Each clinical centre was contacted by phone or email to explain the background of the study, and were requested to confirm their willingness to participate by email. Six of the eleven centres agreed to participate. In addition, a physiotherapist from one of the six CF centres distributed the survey to members of the national physiotherapy CF clinical interest group. The survey was disseminated to a total of 81 HCP.

An individual HCP from each of the six participating CF centres was identified as a source of contact between the researcher and the individual’s MDT. This individual was responsible for circulating the plain language statement and the link to the online survey among their colleagues within their MDT. Participants were asked to provide informed consent at the beginning of the online survey. Data was collected using a web-based survey tool (SurveyMonkey®) between February and March 2019. Ethical approval was granted by Dublin City University Research Ethics Committee (DCUREC/2018/141). Participants were given an initial deadline of 2 weeks from receipt of the first email containing the link to the survey. The deadline was extended for a further two weeks and reminders were sent via email, once per week, to optimize response rate.

### Survey

The 30-item survey was developed by the research team and included questions that were adapted from previous literature in similar chronic conditions investigating i) HCP knowledge and practice of PA prescription in cancer care [18, 19], and ii) the role of person-centered exercise provision within the CF MDT (full survey is included as Additional File 1) [20]. The survey consisted of four sections, which aimed to identify i) the HCP demographic and professional backgrounds ii) their knowledge and practice, and motivators and barriers in relation to PA promotion and prescription and iii) strategies to enhance such prescription in CF care. The format included multiple choice single answer and matrix style questions. Three open ended questions were also included to identify, from the HCP perspective, motivators and strategies to optimize PA prescription to their CF patients.

### Data analysis

Data were analysed using IBM Statistical Package for the Social Sciences (SPSS) version 24. Descriptive statistics, including frequencies, were conducted. Open-ended, free text, data were analysed using thematic analysis [21]. Thematic analysis involved a 6-step process whereby codes were generated and emergent themes were identified, reviewed and defined, ensuring no incidence of overlap to offer a credible and trustworthy interpretation of the participants perceptions. The data were analyzed for all HCP combined and separately for physiotherapists (*n* = 24) and other HCP (*n* = 24).

## Results

Eighty-one HCP received the invitation, and forty-eight HCP participated in the study (59.2% response rate). The respondents demographic and professional characteristics are outlined in Table 1.

**Table 1:** HCP demographic and professional characteristics

Gender	Female	85%
	Male	15%
Age	20-29y	11%
	30-39y	52%
	40-49y	27%
	50-59y	10%
Occupation	Physiotherapist	50%
	Clinical Nurse Specialist	23%
	Registrar	9%
	Dietician	6%
	Consultant	6%
	Surgeon	2%
	Psychologist	2%
	Physician	2%
Years Qualified	0-5y	10%
	6-9y	15%
	10-19y	50%
	>20y	25%
Years in CF care	0-5y	40%
	6-9y	8%
	10-19y	42%
	>20y	10%
Work Setting	Public Hospital	73%
	Specialist Centre	27%
Patient Group	Adult	50%
	Paediatric	27%
	Combined Adult + Paediatric	23%
Pre or Post Transplant	Pre	74%
	Post	26%

### Education regarding PA prescription in CF care

Only 11.6% of HCP reported to have received education in relation to PA prescription for CF populations during their undergraduate studies. There was no great difference between the education received by physiotherapists and other HCP at undergraduate level. Those HCP who graduated during the past 5 years, were no more likely to have received education regarding PA promotion and prescription at undergraduate level than those who graduated > 20 years ago.

Since graduating, three out of every four HCP sought to improve their knowledge of PA prescription for CF populations. Conference attendance, self-directed learning and informal discussion were the three most common sources of further education. Less commonly reported sources of further education included in-service training, workshop or study-day attendance, supervised clinical placement and graduate training by MSc or PhD.

### Knowledge of PA prescription for CF populations

Likert responses indicating HCP level of agreement for having sufficient knowledge of, and familiarity with, the current consensus guidelines are outlined in Table 2. A very high proportion (95%) of physiotherapists agreed or strongly agreed that they had sufficient knowledge about prescribing PA to people with CF, with 85% agreeing or strongly agreeing that they were familiar with the current PA consensus guidelines for people with CF. In contrast, among other HCP 38.1% agreed or strongly agreed to having sufficient knowledge of and familiarity with the consensus guidelines.

**Table 2:** HCP level of agreement for sufficient knowledge of, and familiarity with, the current consensus PA guidelines for CF populations

	Occupation	Strongly Disagree (%)	Disagree (%)	NAND* (%)	Agree (%)	Strongly Agree (%)
**Sufficient Knowledge**	Physiotherapist	0.0	5.0	0.0	60.0	35.0
	Other HCP	4.3	47.8	30.4	17.4	0.0
**Familiarity with Guidelines**	Physiotherapist	0	0	15	40	45
	Other HCP	13	47.8	17.4	17.4	4.3

*NAND Neither agree nor disagree

HCP were asked to describe the current consensus guidelines [16], with respect to the FITT principle criteria, where; F = frequency; the number of times PA should be carried out per week, I = intensity; how hard the individual should be working, T = time; the duration of the PA session and T = type; the mode of activity. Physiotherapists were typically very accurate in their description of the FITT principle criteria with accuracy ranging from 89.5–100%, compared to other HCP who largely reported incorrectly, particularly with respect to the Frequency (15%) and Intensity (10%) components. Table 3 outlines the proportion of participants that correctly described the FITT principle components with respect to the current consensus guidelines.

**Table 3:** HCP accuracy in reporting the current consensus guidelines [14], with respect to the FITT principle criteria

	Physiotherapist	Other HCP
**Frequency (%)**	94.4	15.0
**Intensity (%)**	100.0	10.5
**Time (%)**	94.7	73.7
**Type (%)**	89.5	47.4

### Practice of PA prescription in CF care

There was almost unanimous consensus (94.3%) among the HCP that the physiotherapist, as the PA specialist within the MDT, should take the lead for PA prescription. Among HCP, 80.6% agreed or strongly agreed that recommending PA to their CF patients was part of their professional role. All physiotherapists reported routinely discussing PA at every patient interaction and the majority (81.3%) reported incorporating the current consensus guidelines into their prescription of PA during patient interactions, compared to only 5% of other HCP. Among other HCP, only 42.1% discussed PA at every patient interaction, with the remainder discussing PA at every second patient visit (25%), rarely (8.3%), only at annual review (8.3%) or only when the patient asks (4.2%). The majority of physiotherapists (75%) communicated their PA prescription through a combined method of verbal and written advice, compared to 100% of other HCP communicating this prescription through verbal advice alone.

Half of physiotherapists reported finding it ‘easy’ or ‘extremely easy’ to prescribe PA to their CF patients. The remaining 37.5 and 12.6% of physiotherapists reported finding PA prescription ‘neither easy not difficult’ and ‘difficult’ or ‘extremely difficult’, respectively. Of the other HCP, 30% reported PA prescription to be ‘easy’ or ‘extremely easy’, with 50 and 20% of the remaining HCP finding it ‘neither easy nor difficult’ and ‘difficult’ or ‘extremely difficult’, respectively.

### Motivators

HCP were asked to identify, through an open-ended question, the most prominent motivators that facilitated their PA prescription. Among the most common motivators reported were; improving patient outcomes such as survival rates, pulmonary function, exercise tolerance, psychological well-being and quality of life. The motivators described by HCP are outlined in Table 4.

**Table 4:** HCP motivators for prescribing PA to their CF patients

1. Improving survival rates	
2. Improving pulmonary function	
3. Improving exercise tolerance	
4. Improving psychological well-being	
5. Improving quality of life	

### Barriers

**Fig. 1:**
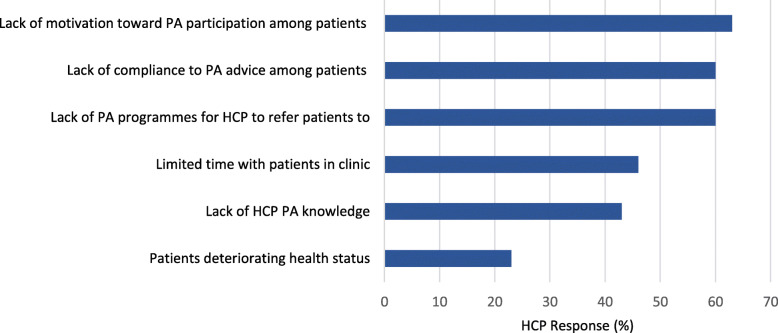
Barriers to HCP prescription of PA to their CF patients. Legend: HCP barriers to PA prescription are listed along the y-axis, with the percentage of respondents along the x-axis

HCP were asked to rank the most prominent barriers to prescribing PA and the results are outlined in Fig. 1. Lack of patient motivation to participate in PA, poor compliance to PA advice given by HCP and a lack of PA programmes to refer patients were the most common barriers reported by all HCP.

### Strategies to optimize PA prescription among HCP

HCP were asked to identify strategies that they believed would improve their ability to prescribe PA to their CF patients. The most common strategies reported by HCP were: i) to recognize one member of the MDT (physiotherapist) as the lead for PA prescription, ensuring that other MDT members are educated and skilled to reinforce this prescription with evidence-based PA promotion, ii) the development of formal, standardized and accredited continuing professional development programmes to improve HCP knowledge surrounding PA prescription and promotion for CF populations, and iii) to increase the development and availability of PA programmes for HCP to refer their CF patients to. The strategies identified by HCP were collated and formed, by the investigator, into statements summarized in Table 5.

**Table 5:** Strategies identified by HCP to optimize their prescription of PA to CF patients

1. Identify one member of the MDT (physiotherapist) as the lead for PA prescription, ensuring other MDT members are educated and skilled to reinforce PA promotion	
2. Improve HCP knowledge surrounding PA prescription and promotion by developing formal, standardized and accredited continuing professional development (CPD) programmes	
3. Increase the development and availability of PA programmes for HCP to refer their CF patients to	

## Discussion

The key findings that emerged from the current study include: i) the lack of education present at undergraduate level with respect to PA promotion and prescription for CF populations, ii) sources of further education sought by the majority of HCP were typically mixed and non-accredited, iii) physiotherapists were more confident in their knowledge and prescription of PA, ensuring use of the guidelines at every patient interaction, compared to other HCP who lacked sufficient knowledge and confidence, and iv) the lack of patient motivation and compliance toward PA advice were among the most common barriers to PA prescription reported by HCP.

The number of HCP who received education with respect to PA prescription for CF populations during their undergraduate degree was very low. This appears not to be specific to CF, as education focusing on PA promotion and prescription for clinical populations in general has been identified as essentially missing from the physiotherapy curriculum in Ireland [22]. This is concerning, given the global movement to transition the healthcare service from primarily focusing on sickness, to a service that focusses on prevention and health promotion. The physiotherapy curriculum in Ireland is primarily focused on impairment, injury and disability, with little time devoted to PA prescription for CF and other chronic disease populations [22]. This is highlighted by the fact that HCP who have graduated during the past 5 years were no more likely to have received education in relation to PA prescription for CF populations during their undergraduate degree than those that graduated 10–19 years ago.

This study has found that HCPs lack confidence in their ability to promote PA, it is a topic that is largely missing from the undergraduate curriculum, and knowledge of certain elements of the guidance could be improved. As a result, we suggest the development of a formal, standardized and structured continuing professional development programme for HCP seeking to improve their knowledge within the area of PA promotion and prescription for CF populations*.* In similar survey studies conducted in the UK and Germany, 100 and 87% of HCP who responded, respectively, indicated that they would benefit from additional CF-specific education, training and resources in relation to PA in CF care [23, 24].

There was unanimous consensus among the HCP that the physiotherapist be identified as the lead, when prescribing PA to patients with CF. This echoes the European CF Society Standards of Care Framework, which states that the specialist CF physiotherapist should take the lead role in delivering high quality treatment, involving physical exercise training [25]. Physiotherapists reported high levels of knowledge and familiarity with the current consensus guidelines, suggesting that they are confident and skilled to adopt this leadership role. Additionally, all physiotherapists reported discussing PA at every patient visit, using a combination of verbal and written PA prescription, largely based on the current consensus guidelines.

Although a significant number of other HCP reported discussing PA with CF patients as part of their professional role, there appeared to be a dearth of knowledge in relation to the appropriate PA prescription for CF populations. This is concerning, as over half of the other HCP actively sought further education to improve their knowledge regarding PA prescription in CF care. Interestingly, the other HCP were unfamiliar with the current consensus guidelines, and they tended to under-prescribe with respect to the *frequency* and *intensity* components of the FITT principle criteria. CF patients receiving this advice may not achieve an appropriate overload stimulus to maintain or improve functional capacity.

When discussing PA, other HCP used verbal advice alone, with only 5% basing their advice on the current consensus guidelines. To ensure optimal patient outcomes, it is important that all HCP within the MDT are working synchronously to effectively communicate the significance of PA to their CF patients [26]. Identifying the physiotherapist as the lead for PA prescription, with other members of the MDT positively reinforcing the benefits of PA through scheduled discussions during clinic visits, will ensure that the patient receives more exposure to PA dialogue, than with the physiotherapist alone. It is evident that other HCP recognize the importance of PA prescription and deem it part of their professional role, yet currently lack the appropriate knowledge to efficiently reinforce PA promotion. This indicates the need for the development of standardized and structured CPD programmes, focusing on patient-centered, evidence-based PA promotion and prescription for CF populations, made available to all HCP working in CF care.

Although HCP are primarily motivated to prescribe PA to improve patient outcomes, they are faced with a number of barriers challenging this prescription. The most common barriers reported by HCP included a lack of compliance and motivation among CF patients to adhere to PA advice. The scarcity of PA programmes for HCP to refer patients was another significant barrier to PA prescription. The development of patient-centered, evidence-based PA programmes, underpinned by theories of behaviour change, would greatly enhance PA prescription for CF HCP. Due to HCP reporting time constraints as a barrier to PA prescription, we suggest that the expert in behaviour change within the MDT, the psychologist, should work in close collaboration with the physiotherapist to implement this.

Wearable technology has the potential to enhance patient motivation and compliance, allowing patients to be more active in their care, and to better understand how their PA behaviours can affect their health in real-time [27]. The adoption of wearable technology, such as activity trackers, and mobile phone applications has the potential to promote patient engagement through personalized PA interventions. Incorporating components of e-health that are convenient and easily accessible for the patient has the potential to change how they engages with healthcare services, and reduce the burden of care and costs associated with healthcare delivery [28]. Previous research suggests that the use of an internet-based program to monitor and encourage PA participation, is both feasible and acceptable among adults with CF [29].

A multifaceted approach is required to address the barriers experienced by HCP regarding PA prescription for CF populations. Investigator-developed recommendations to overcome the aforementioned barriers, and to optimize HCP prescription of PA for CF populations, include i) recognising the physiotherapist as the lead for PA prescription, with other members of the MDT positively reinforcing with appropriate PA promotion, particularly as the patient may not always attend the physiotherapist at every clinic visit, it is critical that a multidisciplinary approach is adopted to ensure regular and consistent discussions around PA are had, ii) the development of recognized, structured and standardized further education and training opportunities to enable HCP to upskill and gain confidence with respect to PA prescription for their CF patients, iii) the introduction of formal in-house educational sessions or workshops, provided by exercise specialists within CF care, to create more awareness around the importance and benefits of PA for CF populations, iv) the development of personalised and evidence-based PA programmes for HCP to refer their patients to, and v) promote the use of components of telemedicine and interventions underpinned by behaviour change theories to employ a patient-centred approach for eliciting positive and sustainable change.

### Limitations

There are limitations within the current study that must be considered when interpreting the results. Firstly, it is important to acknowledge the possible presence of sampling bias. Opportunistic sampling may have resulted in a sample of HCP who recognise the therapeutic impact of PA, overlooking the opinions of those who are not interested in using PA as a therapeutic modality for CF populations. There was also an over-representation of physiotherapists within the current study as a result of the survey invitation being sent to the National Physiotherapy CF Clinical Interest Group and not to other professional clinical interest groups. Also, as the nature the data is self-reported, there is a risk that social desirability bias may have occurred making the results more desirable and portraying a less realistic representation of current knowledge and practice ^12,20^. Clinical exercise physiologists and exercise scientists were not included in this survey as the has not yet been recognized by the Health Service Executive in Ireland.

## Conclusion

Although HCP have the capacity to play an important role in influencing patient PA behaviours, a multi-disciplinary approach is required. We recommend the development of a CPD programme for CF HCP, focusing on enhancing their knowledge and confidence in relation to PA prescription and promotion. Future success will depend on objectively recognising, evaluating and addressing the barriers that are preventing HCP from prescribing and promoting PA to their CF patients. The development of a HCP referral pathway to patient-centred, evidence-based PA programmes that are underpinned by behaviour change may improve patient compliance and motivation towards adherence to PA advice.

## Supplementary Information

**Additional file 1.**

## Data Availability

The datasets used and/or analysed during the current study are available from the corresponding author on reasonable request.

## Abbreviations

PA: Physical activity
CF: Cystic fibrosis
HCP: Healthcare professional
CFTR: Cystic fibrosis transmembrane conductance regulator
MDT: Multi-disciplinary team
SPSS: Statistical package for the social sciences
CPD: Continuing professional development
