# Supplementary material for: Recommendations to improve physical activity prescription for the cystic fibrosis population: an Irish perspective
Source: BMC Health Serv Res. 2020 Nov 19;20:1052. doi: 10.1186/s12913-020-05910-2 (PMC7678110; doi:10.1186/s12913-020-05910-2)
Supplement: Supplementary file 1 — Additional file 1. [file 12913_2020_5910_MOESM1_ESM.pdf]

Dear Health Care Professional,

Thank you for taking the time to complete this questionnaire on exercise prescription in cystic fibrosis.

It should take less than 15 minutes and all of your responses will be anonymous.

Many thanks,
The MedEx Research Team at Dublin City University

Please see the Plain Language Statement (attached to your e-mail invitation to this questionnaire) for all of the details in relation to this study.

Please answer the below questions if happy to do so.

Participation is optional and you can exit the questionnaire at any time by closing this window on your computer.

1. Informed Consent:

- ☐ I have read the Plain Language Statement
- ☐ I understand the information provided
- ☐ I have had the opportunity to ask questions and discuss this study
- ☐ I have received satisfactory answers to all of my questions

Section 1: Your Profile

2. Gender:

- Male
- Female

3. Age range:

- 20 - 29 years
- 30 - 39 years
- 40 - 49 years
- 50 - 59 years
- 60+ years

4. Your occupation:

- Consultant
- General Practitioner
- Surgeon
- Registrar
- Physiotherapist
- Exercise Physiologist
- Research Nurse
- Clinical Nurse Specialist
- Dietician
- Psychologist
- Occupational Therpaist
- Other (please specify)

5. How many years have you been qualified?

- 0 - 5 years
- 6 - 9 years
- 10 - 19 years
- 20+ years

6. How many years of clinical experience do you have working within CF care?

- 0 - 5 years
- 6 - 9 years
- 10 - 19 years
- 20+ years

7. Please indicate your work setting:

- Public Hospital
- Private Hospital
- Specialist CF Centre
- Primary Care Centre
- Community
- Other (please specify)

8. Is your centre:

- Adult
- Paediatric
- Combined

9. Do you specialise in treating patients who are:

- Pre-lung transplant
- Post-lung transplant

10. Please indicate how many CF patients you see in a week (Individual patients, not number of contacts with same patient):

Inpatients:

Outpatients:

[Knowledge]
Education Regarding Exercise in CF Care

The remaining questions relate to physical activity and exercise, which are defined as follows:

Physical Activity:
Physical activity is defined as any bodily movement produced by skeletal muscles that requires energy expenditure. Physical activity includes all forms of activity, such as everyday walking or cycling to get from A to B, active play, work-related activity, active recreation (such as working out in a gym), dancing, gardening or playing active games, as well as organised and competitive sport (World Health Organisation, 2018).

Exercise:
Exercise is a subcategory of physical activity that is planned, structured, repetitive, and purposeful in the sense that the improvement or maintenance of one or more components of physical fitness is the objective (World Health Organisation, 2018)

11. During your undergraduate degree, did you receive educational training in relation to physical activity / exercise prescription specific to people with CF?

- ○ Yes
- ○ No

12. Since qualifying, have you received any information regarding prescribing physical activity / exercise to people with CF?

- ○ Yes
- ○ No

13. If yes, how was this information provided? (Please tick all that apply)

- ☐ Workshop
- ☐ Conference
- ☐ Study day
- ☐ Graduate training (MSc, PhD)
- ☐ In-service training
- ☐ Informal discussion
- ☐ Supervised clinical placement
- ☐ Self-directed learning
- ☐ Other (please specify)

[ ]

14. Please indicate your level of agreement with the following statement:
I have sufficient knowledge about prescribing exercise to people with CF

Strongly Disagree	Disagree	Neither Agree nor Disagree	Agree	Strongly Agree
○	○	○	○	○

15. Please indicate your level of agreement with the following statement:
I am familiar with the current consensus exercise guidelines for people with CF

Strongly Disagree	Disagree	Neither Agree nor Disagree	Agree	Strongly Agree
○	○	○	○	○

16. Can you describe the current consensus exercise guidelines for adults with CF:

Frequency
(how many times per week)

Intensity
(how hard should the individual work)

Time
(how long should the session last)

Type
(what mode of physical activity / exercise)

17. Please indicate your level of agreement with the following statement:
There is a need to improve exercise services available to people with CF

Strongly Disagree	Disagree	Neither Agree nor Disagree	Agree	Strongly Agree
○	○	○	○	○

[Practice]
Exercise Provision within the CF Healthcare Setting

18. Who in your MDT is primarily responsible for discussing exercise training and/or exercise prescription with CF patients?

- ◯ Consultant
- ◯ Registrar / SHO
- ◯ Nurse
- ◯ Physiologist
- ◯ Physiotherapist
- ◯ Dietician
- ◯ Psychologist
- ◯ Social Worker
- ◯ Pharmacist
- ◯ Occupational Therapist
- ◯ Other (please specify)

19. Please indicate your agreement with the following statement:
"Recommending physical activity / exercise to people with CF is part of my role"

Strongly Disagree	Disagree	Neither Agree nor Disagree	Agree	Strongly Agree
◯	◯	◯	◯	◯

20. Please indicate your level of agreement with the following statement:
"I would be confident in appropriately prescribing physical activity / exercise programmes to my CF patients"

Strongly Disagree	Disagree	Neither Agree nor Disagree	Agree	Strongly Agree
◯	◯	◯	◯	◯

21. Do you routinely discuss physical activity / exercise with your CF patients?

- ◯ Yes, at every visit
- ◯ Yes, regularly (1 in every 2 visits)
- ◯ Rarely
- ◯ Only at annual review
- ◯ Only when the patient asks
- ◯ Never

22. If yes, is this advice:

- ◯ Written
- ◯ Verbal
- ◯ Both

23. Please specify what advice is given to patients?

24. Is this advice based on the guidelines?

- ◯ No, not based on guidelines
- ◯ Yes, based on guidelines: (Please provide guidelines used)

25. Please estimate what % of your CF patients you recommended physical activity/exercise to in the past 6 months?

- ☐ <10%
- ☐ 10 - 25%
- ☐ 25 - 50%
- ☐ 50 - 75%
- ☐ 75 - 100%

26. How difficult, in your opinion, is it to include prescription of physical activity / exercise into appointments with your CF patients?

Extremely Difficult	Difficult	Neither Difficult nor Easy	Easy	Extremely Easy
◯	◯	◯	◯	◯

27. What are the most prominent barriers that you face in relation to prescribing exercise to your CF patients? *(Please rank your top 3 in order from the list below: 1 = most significant barrier)*

- [ ] Limited time with patients
- [ ] Poor physical activity compliance among CF patients
- [ ] Patients' health status deems physical activity/exercise promotion inappropriate
- [ ] Lack of motivation/interest from CF patients in relation to physical activity/exercise
- [ ] Lack of knowledge regarding physical activity/exercise prescription for people with CF
- [ ] Lack of exercise rehabilitation programmes to refer to

Please note, for the final 3 questions in this section, responses in the form of bullet points, short sentences and paragraphs are all welcome

28. What motivates you/would motivate you to prescribe exercise to CF patients? (Please provide as much detail as possible)

29. What strategies do you think would optimise healthcare professionals' **prescription of exercise** to CF patients? (Please provide as much detail as possible)

30. What strategies do you think would optimise **adherence** of CF patients to prescribed exercise programmes? (Please provide as much detail as possible)
